# Age-Dependent Neuroendocrine Signaling from Sensory Neurons Modulates the Effect of Dietary Restriction on Longevity of *Caenorhabditis elegans*

**DOI:** 10.1371/journal.pgen.1006544

**Published:** 2017-01-20

**Authors:** Marissa Fletcher, Dennis H. Kim

**Affiliations:** Department of Biology, Massachusetts Institute of Technology, Cambridge, MA, United States of America; University of California San Francisco, UNITED STATES

## Abstract

Dietary restriction extends lifespan in evolutionarily diverse animals. A role for the sensory nervous system in dietary restriction has been established in *Drosophila* and *Caenorhabditis elegans*, but little is known about how neuroendocrine signals influence the effects of dietary restriction on longevity. Here, we show that DAF-7/TGFβ, which is secreted from the *C*. *elegans* amphid, promotes lifespan extension in response to dietary restriction in *C*. *elegans*. DAF-7 produced by the ASI pair of sensory neurons acts on DAF-1/TGFβ receptors expressed on interneurons to inhibit the co-SMAD DAF-3. We find that increased activity of DAF-3 in the presence of diminished or deleted DAF-7 activity abrogates lifespan extension conferred by dietary restriction. We also observe that DAF-7 expression is dynamic during the lifespan of *C*. *elegans*, with a marked decrease in DAF-7 levels as animals age during adulthood. We show that this age-dependent diminished expression contributes to the reduced sensitivity of aging animals to the effects of dietary restriction. DAF-7 signaling is a pivotal regulator of metabolism and food-dependent behavior, and our studies establish a molecular link between the neuroendocrine physiology of *C*. *elegans* and the process by which dietary restriction can extend lifespan.

## Author Summary

Reductions in food intake have long been observed to improve longevity, extending lifespan in many evolutionarily divergent organisms. While great progress has been made in identifying the mechanisms by which nutritional interventions act to delay the aging process, much remains unclear. Particularly, while work in multiple species has found evidence that the sensation of food availability by the nervous system contributes to lifespan extension in response to reduced food levels, little is known about how these contributions are executed. Here, we have characterized how a specific neuroendocrine peptide, expressed in a set of sensory neurons, responds to changes in food conditions to modulate lifespan effects of dietary restriction at the organismal level. We further find that age-related changes in expression of this neuroendocrine signal contribute to the declining efficacy of nutritional interventions as animals get older. This work highlights the importance of neuroendocrine regulation in both the aging process and in treatments aimed at increasing longevity.

## Introduction

Adult reduction in caloric intake and restriction of feeding periods have been shown to substantially increase lifespan across evolutionarily diverse organisms [1,2]. Collectively, such treatments have been referred to as dietary restriction (DR). DR has been shown to be effective even when initiated in later phases of adult life, although the efficacy of the treatment has been observed to diminish with advancing age in *Caenorhabditis elegans* [3,4]. Genetic studies in *C*. *elegans* have defined roles for mediators of stress response pathways, such as DAF-16/FoxO, PHA-4/FoxA and SKN-1/Nrf2, as well as the intracellular energy sensors TOR and AMPK in mediating the effects of DR on longevity [5–8]. Other studies have suggested that external cues are also critical in eliciting a DR response that extends lifespan in *C*. *elegans* [9]. In both *C*. *elegans* and *Drosophila*, the efficacy of DR treatment can be abrogated by the addition of food odors, and longevity in *Drosophila* can be extended by reduction of olfactory function [10,11]. Similarly, studies in *C*. *elegans* have shown that mutation of genes implicated in sensory systems or ablation of chemosensory neurons results in extended lifespan [12–14]. Specifically, a pair of gustatory neurons in *C*. *elegans*, the ASI neuron pair, have been shown to be required for lifespan extension in response to dietary restriction [6].

In the present study, we sought to explore the signaling mechanisms by which perceptions in the nervous system of food availability contribute to the DR response in peripheral tissues. We have focused our attention on the gene *daf-7*, which encodes a TGFβ ligand that is secreted from the ASI neurons to control diverse behaviors of *C*. *elegans* [15–18]. DAF-7 has previously been implicated in longevity and food sensing; *daf-7* mutant animals are reported to be long-lived in a manner that is dependent on food levels and also exhibit defects in adjusting feeding behaviors in response to periods of starvation [19–21]. However, the role of DAF-7 in lifespan extension in response to DR has not been fully investigated.

Here, we have focused on expanding understanding of the role that the DAF-7 signaling pathway has in lifespan extension in response to limited nutrient availability. We have determined that DAF-7 is a key neuroendocrine signal required in the ASI neurons for response to dietary restriction. Moreover, we find that age-related changes in *daf-7* expression contribute to the reduced sensitivity that older animals have to DR treatment, suggesting that the efficacy of DR interventions that delay aging can be modulated by neuroendocrine signaling.

## Results

### Neuronal DAF-7/TGFβ signaling promotes lifespan extension in response to dietary restriction

**Fig 1:**
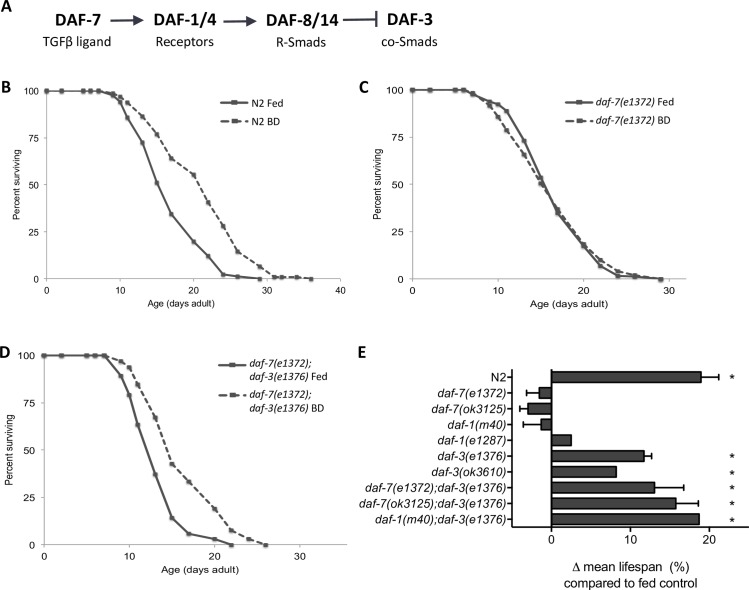
The DAF-7 signaling pathway is required for lifespan extension in response to dietary restriction. A) Summary of the DAF-7/TGFβ pathway B-D) Representative lifespan curves of N2 (B), *daf-7(e1372)* (C), and *daf-7(e1372);daf-3(e1376)* (D) animals subjected to control (fed, solid lines) or bacterial deprivation (BD, dashed lines) diets. E) Summary of all alleles tested for BD response. * indicates BD lifespan was significantly different (p ≤ 0.001) than fed control group in all experiments, error bars reflect SEM. See S2 Table for individual experiment details.

We investigated the role of the DAF-7/TGFβ pathway in lifespan extension in response to dietary restriction using the bacterial deprivation (BD) method, where animals are moved to solid media completely lacking a bacterial food source during adulthood [3,4]. Using this protocol (see Methods for details) at 25°C and initiating BD treatment at day 3 of adulthood, we observed an average 19.5% extension of mean lifespan in wild-type animals, comparable to what has been reported previously when taking into account changes in experimental temperature (Fig 1B and 1E; S2 Table). Using multiple loss-of-function alleles, we observed that mutations in the *daf-7* gene, encoding a TGFβ family ligand, or in the *daf-1* gene, encoding the Type I TGFβ receptor, abrogated the lifespan extension conferred by BD (Fig 1C and 1E; S2 Table). This is consistent with a prior report which found that *daf-7* mutant animals are resistant to longevity fluctuations due to altered food levels [21]. We observed that the strong dependence of lifespan extension conferred by BD on DAF-7 was temperature dependent, as *daf-7* mutant animals retained lifespan extension, albeit reduced relative to wild type, when propagated 20°C (S2 Table), as reported previously [22].

Different regimens of dietary restriction have been found to extend lifespan in *C*. *elegans* through separate genetic pathways [23]. To ensure the effects we observed were not an outcome specific to the BD method of DR, we also tested *daf-7* pathway mutants in a second, distinct protocol for dietary restriction, referred to as solid dietary restriction (sDR), in which adult animals are exposed to a diluted bacterial food source that is refreshed every other day [8]. Using the sDR method, we observed results consistent with our BD data, where mutants in either *daf-7* or *daf-1* have diminished lifespan extension in response to sDR (S1 Fig; S3 Table). DAF-7 signaling through DAF-1 has been shown to act through inhibition of the co-SMAD DAF-3 (Fig 1A) [24,25]. We found that *daf-3* mutation could suppress the loss of sensitivity to dietary restriction observed in *daf-7* and *daf-1* mutants (Fig 1D and 1E, S1 Fig; S2 and S3 Tables).

Mutations in *daf-7* have previously been observed to result in phenotypes such as diminished pumping, increased dauer entry, and increased fat storage [17]. Genetic analysis of the individual phenotypes of *daf-7* mutant animals has identified distinct downstream genetic pathways that act to mediate each of these DAF-7-dependent phenotypes [17], enabling us to determine if any of these pleiotropies might be associated with the diminished ability of DAF-7 pathway mutants to respond to DR. The pumping defect of *daf-7* mutants is small in magnitude compared to the decrease in pharyngeal pumping observed in feeding-defective *eat* mutants that are used as genetic models of DR [26,27]. Nonetheless, to test this possibility, we determined the effects of combining a *daf-1* mutation with mutations in *tbh-1* and *tdc-1*, which have been shown to suppress the feeding rate changes in *daf-1* and *daf-7* mutants [17]. To determine if signaling through pathways promoting dauer formation might be involved in the DR phenotype, we examined a *daf-1;daf-12* double mutant. To determine if fat storage might be contributing to the DR defects we observed, we constructed *daf-1 mgl-3;mgl-1* mutants, in which fat storage increases arising from diminished DAF-7 signaling are specifically suppressed [17]. None of these secondary mutations were able to suppress the BD defect of *daf-1* mutant animals, decoupling these three phenotypes from the DR response that is dependent on DAF-7 signaling (S2 Fig; S2 Table).

**Fig 2:**
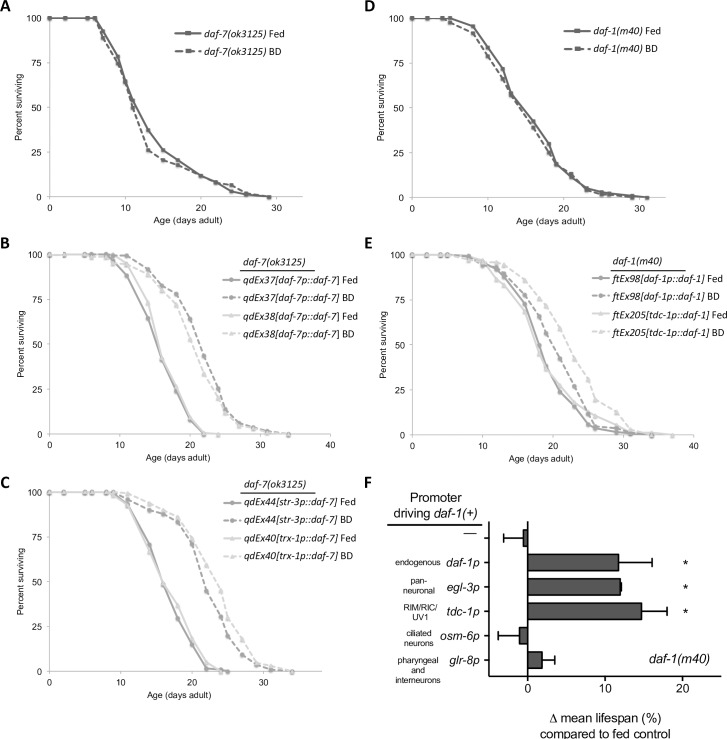
DAF-7 originating from chemosensory neurons acts on RIM/RIC interneurons to promote lifespan extension in response to DR. A-C) Lifespan curves displaying rescue of BD defect of *daf-7(ok3125)* animals (A) by reintroducing wild-type *daf-7* under the endogenous promoter (B), the ASI specific *str-3* promoter, or the ASJ specific *trx-1* promoter (C). D-E) Representative curves displaying rescue of BD defect of *daf-1(m40)* animals (D) by expressing wild-type *daf-1* under its own promoter or the RIM/RIC/UV1 specific promoter, *tdc-1* (E). F) Summary of all *daf-1(m40)* rescue experiments performed. * indicates BD lifespan was significantly different (p ≤ 0.05) than fed control group in all experiments, error bars reflect SEM. See S2 Table for individual experiment details.

Prior studies established that *daf-7* is expressed principally in the ASI neuron pair, but also in additional sensory neurons when *C*. *elegans* is propagated on *E*. *coli* bacterial food, and that *daf-7* expression is induced in the ASJ neuron pair upon exposure to metabolites of *Pseudomonas aeruginosa* [15,16,18]. We found that reintroducing wild-type *daf-7* into *daf-7(ok3125)* mutants rescued the BD defect of these animals (Fig 2A and 2B). Additionally, *daf-7(+)* driven by ASI or ASJ specific promoters was also sufficient to rescue the BD defect of *daf-7* mutant animals, consistent with the secretory nature of the DAF-7 ligand (Fig 2C). Unlike the expression of the DAF-7 ligand, the DAF-1 receptor is broadly expressed in the *C*. *elegans* nervous system [25,28]. To determine the functional targets receiving DAF-7 signal, we examined the ability of *daf-1(m40)* animals to respond to DR when a wild-type *daf-1* transgene had been expressed in different subsets of cell types under heterologous promoters [17]. *daf-1* expression in the nervous system was sufficient to restore lifespan extension in response to BD. Furthermore, as has been demonstrated for other *daf-7* regulated phenotypes [17], we observed that the RIM/RIC interneurons are the specific sites of action for the *daf-1* receptor for lifespan extension in response to BD treatment (Fig 2D–2F).

### Bacterial deprivation acutely induces expression of *daf-7* in the ASI neurons of adult animals

**Fig 3:**
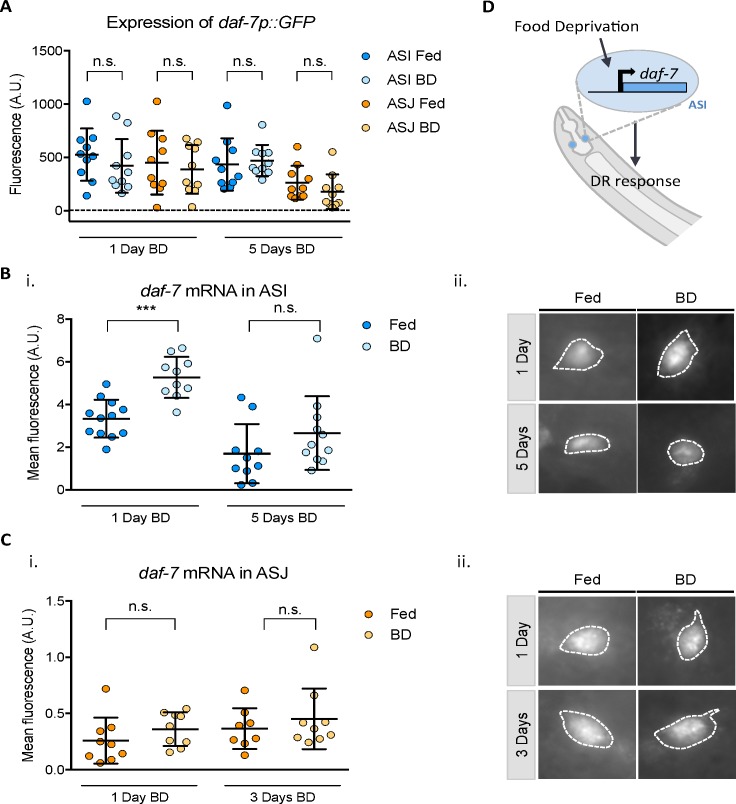
Dietary restriction acutely increases *daf-7* expression in adult animals. A) Quantification of GFP expression driven by *ksIs2[daf-7p*::*GFP]*, normalized to exposure time in fed and BD conditions. Representative of 3 replicates performed with n>10 animals per condition. B,C) (i.) Quantification of fluorescence of FISH probes designed against *daf-7* in ASI (identified by co-localization with *str-3p*::*GFP*) (B) and ASJ (identified by co-localization with *trx-1p*::*GFP*) (C) under fed and BD conditions. *** represents p < 0.001 by unpaired t-test. (ii.) Representative images of ASI or ASJ neurons corresponding to the quantifications presented in (i). All images taken with the same exposure time. D) Model of *daf-7* expression change in response to dietary restriction.

Given the results of our genetic analysis of the DAF-7 signaling pathway in dietary restriction, we sought to examine how *daf-7* expression might change in response to DR intervention. We were unable to detect a change in expression using quantification of the transcriptional reporter, *ksIs2[daf-7p*::*GFP]*, in fed versus BD treated animals (Fig 3A). We have previously observed that fluorescent in situ hybridization (FISH) provides more precise kinetic resolution of the dynamics of *daf-7* transcription than does the *ksIs2* GFP reporter [18]. By performing FISH on animals subjected to BD, we were able to detect a slight but consistent upregulation of *daf-7* mRNA transcription. Worms exhibited an increase in *daf-*7 mRNA in ASI neurons in animals fixed 24 hours after BD treatment was initiated, but no detectable difference was found after a period of 5 days had passed (Fig 3B). Of note, we observed that aging adult animals began to exhibit low-level expression in the ASJ neurons, but we did not observe any changes in *daf-7* mRNA in the ASJ neurons in response to BD (Fig 3A and 3C). These data suggest that in response to food deprivation, *daf-7* transcription is acutely activated in the ASI neuron pair, which promotes lifespan extension mediated by DR (Fig 3D).

### DAF-7 is required for intestinal DAF-16/FoxO translocation in response to food deprivation

**Fig 4:**
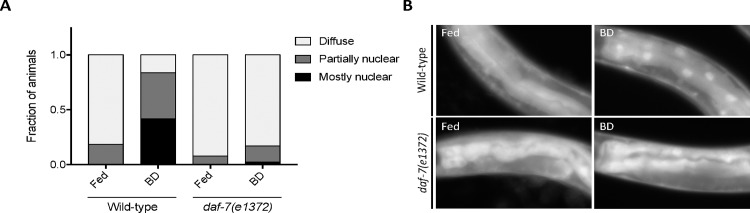
*daf-7* is required for DAF-16/FoxO nuclear translocation in response to BD. A) State of DAF-16a/b::GFP localization pattern in fed versus BD conditions. Representative of 2 replicates with total n = 116–154 animals per condition. B) Representative images of the *zIs356[daf-16p*::*daf-16a/b*::*GFP]* reporter in the intestine of wild-type or *daf-7(e1372)* animals in fed and BD conditions.

In response to food cues, neuroendocrine signals originating from chemosensory neurons can influence the activity of DAF-16/FoxO in the intestine [29,30]. To determine if DAF-7 signaling contributes to the DR response via DAF-16/FoxO activation, we monitored the localization of the *zIs356[daf-16p*::*daf-16*::*GFP]* transgene in wild-type and *daf-7* mutant backgrounds. In response to food deprivation, wild-type animals shift from mostly cytosolic to nuclear localized DAF-16::GFP [29]. A *daf-7* loss-of-function mutation abrogated this intestinal DAF-16::GFP translocation in BD conditions compared to wild-type animals (Fig 4). These data were surprising particularly considering that DAF-16 activation has been implicated in the setting of *daf-7* loss-of-function [31]. However, we note that consistent with reports by others [19], we did observe an increase in nuclear DAF-16::GFP in the *daf-7(e1372)* background in other tissues such as the muscle and hypodermis in both fed and BD conditions (S3 Fig). This observation suggests that specifically in response to BD, an increase in *daf-7* expression stimulates activation of DAF-16 in the intestine, which helps to promote longevity. This model is fitting with prior reports that have implicated a role for DAF-16/FoxO in mediating lifespan extension in response to various forms of DR [8,23] and in food sensing mutants [12].

### Decline of *daf-7* expression in aging animals reduces organismal sensitivity to dietary restriction

**Fig 5:**
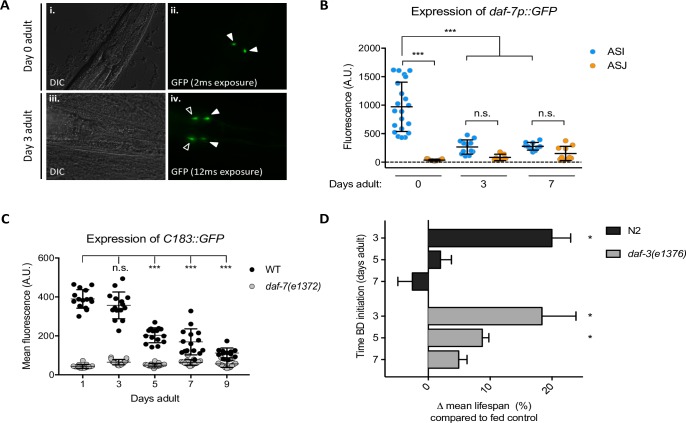
*daf-7* expression declines in aging animals. A) Expression pattern of *ksIs2[daf-7p*::*GFP]* reporter in young (i,ii) and aged (iii,iv) animals. Solid triangles indicate ASI neurons, open triangles indicate ASJ neurons. B) Quantification of GFP expression driven by *ksIs2[daf-7p*::*GFP]*, normalized to exposure time. Representative of 4 replicates with n>10 animals per day. *** represents p < 0.001 by one-way ANOVA C) Quantification of GFP expression driven by *cuIs5[C183*::*myo-2p*::*GFP]* in aging animals, normalized to exposure time. WT statistics indicated on graph, *daf-7(e1372)* statistics- Day 3 p = 0.0002; Day 5 p = 0.5225; Day 7 p = 0.0005; Day 9 p = 0.0445; all differences in *daf-7(e1372)* are a result of increased GFP fluorescence later in life. Representative of 4 replicates with n>10 animals per genotype per day. *** represents p < 0.001 by one-way ANOVA D) Summary of lifespan experiments initiating BD at various times in adulthood. * indicates BD lifespan was significantly different (p < 0.005) than fed control group in all experiments. See S4 Table for individual experiment details.

We measured *daf-7* expression as animals aged during adulthood using the *ksIs2[daf-7p*::*GFP]* reporter strain. We observed that *daf-7* expression is maintained throughout the life of adult animals in the ASI neurons. As noted above, we also observed *daf-7* expression in the ASJ neuron pair as animals age, with all animals exhibiting ASJ expression by day 3 of adulthood (Fig 5A). In contrast to the marked induction of *daf-7* expression in both ASI and ASJ neurons in response to *P*. *aerugionsa* [18], in aging animals, *daf-7* expression in ASJ remained relatively low (Fig 5B). Moreover, we observed that *daf-7* expression in the ASI neuron pair significantly decreased with age (Fig 5B). We confirmed these findings by FISH using probes targeted to endogenous *daf-7* mRNA to eliminate the possibility that these observations were an artifact of using a transgenic reporter. Our FISH results support our observations of the *ksIs2* GFP reporter strain. ASI neurons from aged animals show decreased *daf-7* expression; and while there is no detectable *daf-7* mRNA in ASJ neurons of young animals, we were able to observe *daf-7* mRNA in older adults (S4 Fig).

We sought to corroborate these changes in *daf-7* expression in these sensory neurons with a measure of how much functional DAF-7 was secreted, so we utilized the *cuIs5[C183*::*GFP]* reporter of DAF-3 activity. DAF-3 negatively regulates *C183* enhancer activity *in vivo*, resulting in low GFP fluorescence when DAF-3 is active [32]. The transgenic *cuIs5[C183*::*GFP]* reporter provides a measure of DAF-7 signal production by examining the downstream effects on DAF-3 in a neighboring tissue. We found that GFP fluorescence was diminished in an age-related, DAF-7-dependent manner, consistent with less overall DAF-7 signaling in aging worms (Fig 5C).

In addition to experiencing declines in healthspan indicators such as feeding rate and mobility, aging worms also become diminished in their ability to respond to dietary restriction treatment to extend lifespan [3,33]. We wondered if part of the insensitivity older animals have to dietary restriction treatment could be attributed to diminished levels of DAF-7 that cause an increased amount of DAF-3 activity that blocks responses leading to lifespan extension in response to DR in aging animals. To test this hypothesis, we conducted BD experiments where BD treatment was initiated at multiple time points, beginning on days 1, 3, 5 or 7 of adulthood, in wild-type or *daf-3* mutant animals. We found that wild-type animals experience a robust lifespan extension when BD was began on days 1 or 3, but were unable to respond when BD was started on days 5 or 7 (Fig 5D, S4 Table), consistent with prior studies [3]. By contrast, *daf-3* mutant animals were able to maintain the ability to respond to BD on day 5 (Fig 5D), suggesting that age related decline in the ability to respond to dietary restriction can be attributed, in part, to increased DAF-3 activation as a result in diminished *daf-7* expression. Additionally, animals overexpressing *daf-7* retain the ability to respond to BD and extend lifespan late in life at a time when wild type animals no longer exhibit lifespan extension in response to BD (S5 Fig).

## Discussion

**Fig 6:**
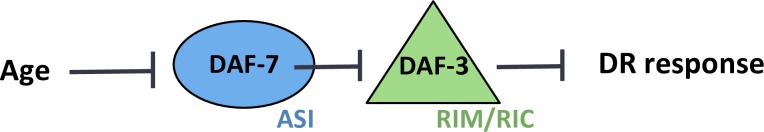
A decline in neuronal *daf-7* expression with advancing age alters sensitivity to effects of DR on lifespan. Declines in *daf-7* expression in ASI chemosensory neurons with age inhibit activity of *daf-3* in RIM/RIC interneurons, the active form of which is able to interfere with the DR response in other tissues.

DAF-7 is at the nexus of feeding behaviors and fat metabolism [17,20], suggestive of neuroendocrine links between the nervous system and secondary tissues. We have described how neuroendocrine signaling through the DAF-7/TGFβ pathway is required for lifespan extension in response to DR in *C*. *elegans*. Whereas canonical energy sensing pathways, such as AMPK and TOR, have been shown to be involved in lifespan extension in response to DR, the role of neural regulation by sensory systems of the DR response is less understood [1,10,11]. Prior studies have established the ASI neuron pair as a cell non-autonomous regulator of the DR response, identifying the insulin-like peptide INS-6 and the SKN-1/Nrf2 transcription factor as relevant agents in initiating communication to downstream cells and tissues [6,29]. We have shown that in response to DR, the ASI pair also secretes the neuroendocrine ligand, DAF-7, which signals to the RIM/RIC interneurons to suppress the co-SMAD DAF-3. In the absence of negative regulation by DAF-7, increased DAF-3 activity blocks the lifespan extension caused by DR (Fig 6).

In the developing animal, the DAF-7 ligand is produced in favorable conditions that promote entry into reproductive development, specifically in the presence rather than the absence of bacterial food [15,16]. Our data are suggestive of an acute increase in *daf-7* expression in the ASI neuron pair in response to the withdrawal of bacterial food, indicating that the dynamic expression of *daf-7* of developing larvae may differ from that of adult animals in response to changing environmental conditions such as DR treatments. Indeed, while bacterial deprivation extends the lifespan of adult animals, the introduction of DR-like treatments in young larvae either prompts entry into the dauer state or has detrimental effects on developing animals that have already surpassed the dauer decision checkpoint [34,35].

Whereas a recent study showed that adult animals exposed to diminishing amounts of bacterial food exhibit decreased *daf-7* expression in the ASI neurons after a period of four days [21], our data, recording levels of *daf-7* mRNA using FISH-based detection at multiple time points after the complete withdrawal of food, reveal a complex relationship pattern of dynamic *daf-7* expression in the ASI neurons of adult animals in response to the withdrawal of bacterial food. We observe an initial increase in *daf-7* expression in animals subjected to BD conditions, consistent with our genetic data implicating a requirement for DAF-3 inhibition for lifespan extension in response to BD. We observe that at later times following the withdrawal of bacterial food, *daf-7* expression is maintained relative to initial levels of expression, in marked contrast to what has been observed when developing larvae are subjected to starvation conditions [15].

Our study builds upon previous observations that have linked the *daf-7* gene with aging and the influence changing food levels has on longevity [19,21]. Together, our genetic findings and expression analyses support a model where active DAF-3 is sufficient to disrupt the animals’ sensory abilities and prevent lifespan extension in response to DR (Fig 6). Because *daf-3* mutant worms are capable of responding to DR, the DAF-7 signaling pathway does not seem to have a direct role in altering metabolism in other tissues to extend lifespan in response to limited food levels. Rather, DAF-7 secreted by the chemosensory neurons seems to be a key neuroendocrine signal that allows animals to properly sense reductions in nutrient availability, which eventually results in activation of DAF-16/FoxO in the intestine under food deprivation. Moreover, our data suggest that an age-dependent decline in neuronal *daf-7* expression also underlies the diminished sensitivity of aging animals to the lifespan effects of DR, linking a decline in neuroendocrine function to the loss of DR efficacy with advancing age.

In human aging, decline in olfactory function is one of the largest predictors of mortality- a stronger independent risk factor for death than causes such as cancer or heart failure [36]. Our study suggests that the modulation of a specific neuroendocrine signaling pathway active in chemosensory neurons involved in the sensation of bacterial food may alter the sensitivity of *C*. *elegans* to the effects of DR. We speculate that therapeutic strategies targeting analogous neuroendocrine pathways in mammals may be able to function in concert with dietary modifications to promote longevity.

## Materials and Methods

### *C*. *elegans* strains

*C*. *elegans* were maintained at 16°C on *E*. *coli* OP50 as previously described [37]. For a list of all strains used in this study, see S1 Table.

### Lifespan assays

Due to the egg-laying defect of *daf-7* pathway mutant animals, synchronized populations were prepared by egg-prep of gravid adult worms in bleach followed by L1 arrest overnight in M9 buffer. L1s were placed on OP50 seeded Nematode Growth Media (NGM) plates and raised to the L4 larval stage at 16°C. Upon reaching L4, worms were transferred onto NGM plates containing 12 μM FUDR (to prevent matricidal effects of *daf-7* pathway mutants as well as progeny production) and 0.01 mg ampicillin seeded with 10X concentrated OP50 from an overnight culture and moved to 25°C (to avoid AVID [38] as well as enhance *daf-7* mutant phenotypes [15,39]). Unless otherwise noted, on day 3 of adulthood (where day 0 is defined as L4 stage), worms were transferred to either fed or DR conditions on NGM plates made without peptone to prevent bacterial growth and rimmed with 150 μL of 10 mg/mL palmitic acid to prevent worms from crawling off the plates. For BD experiments, fed plates were seeded with 200 μL of 10X concentrated OP50 from an overnight culture and BD plates were unseeded. For sDR experiments, fed plates were seeded with 200 μL of OP50 at a concentration of 2x10^10^ bacteria/mL and sDR plates with 200 μL of OP50 at 5x10^8^ bacteria/mL. At least 2 plates per condition were used in all experiments. Worms were scored for death (defined as failure to respond to prodding with a platinum wire) every 1–3 days beginning around day 4 of adulthood. Animals exhibiting vulval rupture were censored. Worms that crawled off the plate were never considered. Representative experiments are presented here. For lifespan statistics of individual experiments, see S2–S4 Tables.

### GFP expression experiments

Synchronized populations were prepared as above and treated in the same manner as worms subjected to lifespan analysis (raised to L4 16°C, then shifted to ampicillin/FUDR plates and placed at 25°C). Animals were examined for GFP fluorescence on the indicated days. All images were acquired with an Axioimager Z1 microscope using animals mounted on glass slides, anesthetized by 100mM sodium azide. Quantification of *daf-7p*::*GFP* was performed by taking the maximum intensity by FIJI software [40] within the ASI or ASJ neuron at 40X magnification. Quantification of *C183*::*GFP* was done by taking the average intensity by FIJI software [40] within the entire pharynx at 20X magnification. All quantifications were normalized by exposure time and background fluorescence (measured individually for each image). Day 3 adult *zIs356[daf-16p*::*daf-16a/b*::*GFP]* strains were examined on a fluorescent dissecting microscope after 4 hours of bacterial deprivation. Representative images were taken at 20X magnification. Two to four replicates were performed for all experiments presented.

### Fluorescent in situ hybridization

Synchronized populations were established as above. FISH was performed as previously described [41]. At the indicated times and treatments, animals were washed twice with M9 buffer before fixation with 4% formaldehyde at room temperature, followed by PBS washes and suspension in 70% RNase free ethanol and stored at 4°C. To image, all samples from an individual experiment were incubated overnight with FISH probes designed against *daf-7* mRNA (coupled to Cy5 dye) [18] in hybridization solution at 30°C. The next day, animals were imaged with a Nikon Eclipse Ti Inverted Microscope outfitted with a Princeton Instruments PIXIS 1024 camera. A GFP marker was used to focus on the neuron of interest and obtain a single image using a Cy5 filter. This method of image acquisition does not allow resolution of single mRNA molecules, thus quantification of *daf-7* was done using FIJI software [40] to outline either ASI or ASJ and obtaining the mean intensity and subtracting background fluorescence (measured by obtaining the mean intensity of a small space immediately adjacent to the neuron being quantified). A minimum of 2 replicates was performed for all experiments presented.

### Statistical analysis

The log-rank statistical test was used to determine *p*-values for lifespans. Using Graphpad Prism, an unpaired t-test, one-sample t-test, or one-way ANOVA was used to determine significance in quantification of expression experiments.

## Supporting Information

S1 Fig *daf-7* signaling is required for additional forms of dietary restriction. **A-E)** Representative curves of animals subjected to control fed (solid lines) or sDR (dashed lines) dietary regimens. **F)** Summary of all sDR experiments. * indicates BD lifespan was significantly different (p ≤ 0.001) than fed control group in all experiments, error bars reflect SEM. See S3 Table for individual experiment details.
(TIF)

S2 Fig Feeding behavior, dauer formation, and fat storage phenotypes of *daf-7* pathway mutants can be uncoupled from the lack of lifespan extension in response to BD. Summary of lifespan experiments suppressing specific phenotypes caused by increased *daf-3* activity. Introducing suppressor mutations in genes controlling changes to feeding rate (*tdc-1* and *tbh-1*), dauer formation (*daf-12*), and fat storage (*mgl-3;mgl-1*) was insufficient to restore lifespan extension by BD in *daf-1(m40)* mutant animals. * indicates BD lifespan was significantly different (p ≤ 0.001) than fed control group in all experiments, error bars reflect SEM. See S2 Table for individual experiment details.
(TIF)

S3 Fig *daf-7(e1372)* mutants have increased basal nuclear DAF-16::GFP. Representative images of the *zIs356[daf-16p*::*daf-16a/b*::*GFP]* reporter in muscle cells of BD treated wild-type or *daf-7(e1372)* animals in fed and BD conditions.
(TIF)

S4 Fig FISH of *daf-7* mRNA. Images and quantification of fluorescence of FISH probes designed against *daf-7* in ASI (identified by co-localization with *str-3p*::*GFP*) (A,B) and ASJ (identified by co-localization with *trx-1p*::*GFP*) (C,D) in young and aged animals. (B) * indicates p < 0.05 by unpaired t-test. (D) * indicates p < 0.05 significantly different from zero by one-sample t-test.
(TIF)

S5 Fig *daf-7* overexpression extends the DR response window. Summary of lifespan experiments in two rescue lines overexpressing *daf-7*. * indicates BD lifespan was significantly different (p ≤ 0.001) than fed control group in all experiments, error bars reflect SEM. See S4 Table for individual experiment details.
(TIF)

S1 Table Strain list. (XLSX)

S2 Table BD lifespan data. Results for individual lifespan assays with BD treatment.
(XLSX)

S3 Table sDR lifespan data. Results for individual lifespan assays with sDR treatment.
(XLSX)

S4 Table Late BD lifespan data. Results for individual lifespan assays where BD treatment was initiated at multiple ages.
(XLSX)
