# Supplementary figures and images for: Age-Dependent Neuroendocrine Signaling from Sensory Neurons Modulates the Effect of Dietary Restriction on Longevity of Caenorhabditis elegans
Source: PLoS Genet. 2017 Jan 20;13(1):e1006544. doi: 10.1371/journal.pgen.1006544 (PMC5291536; doi:10.1371/journal.pgen.1006544)

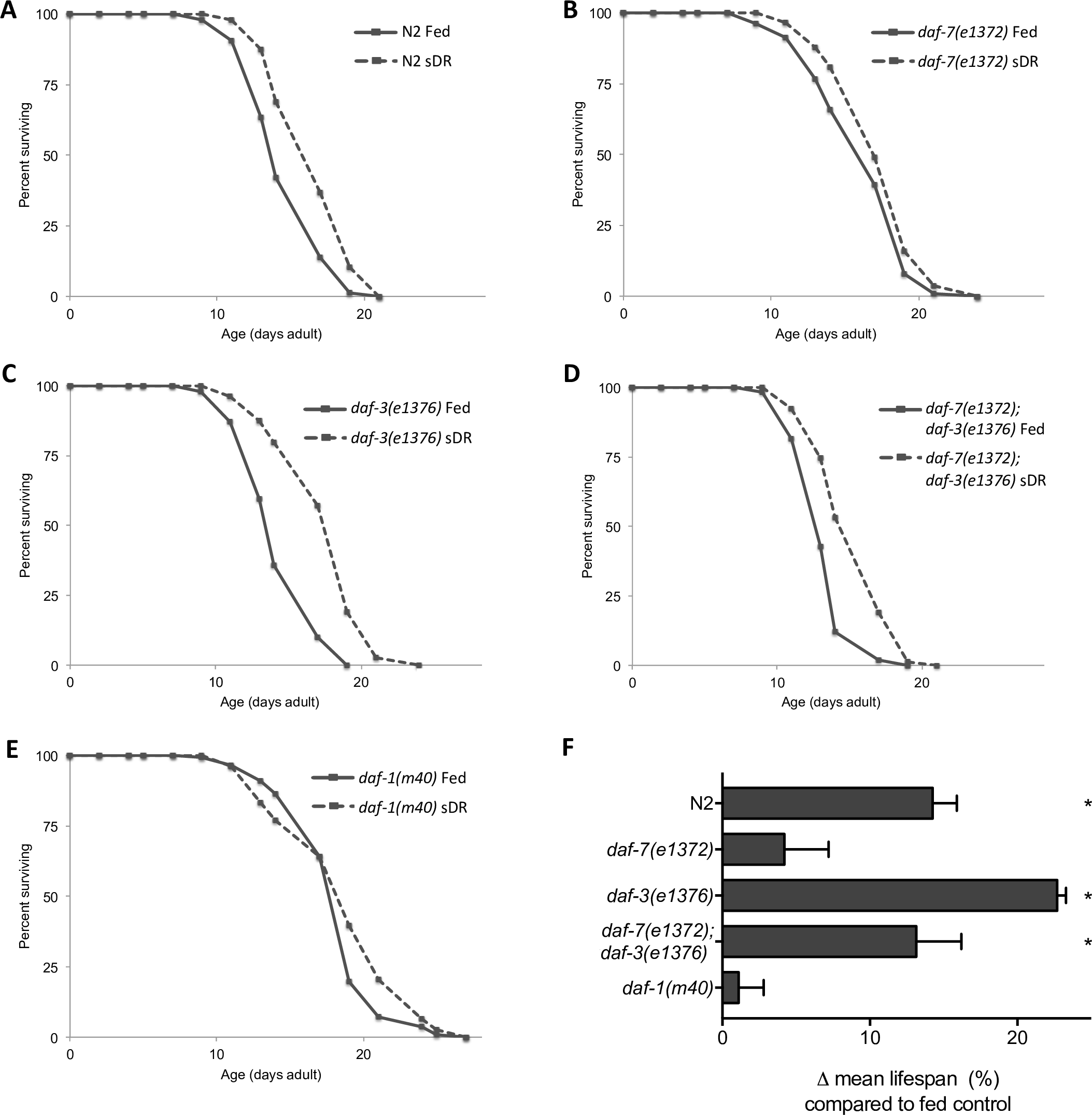

Supplement: S1 Fig — A-E) Representative curves of animals subjected to control fed (solid lines) or sDR (dashed lines) dietary regimens. F) Summary of all sDR experiments. * indicates BD lifespan was significantly different (p ≤ 0.001) than fed control group in all experiments, error bars reflect SEM. See S3 Table for individual experiment details. (TIF) [file pgen.1006544.s001.tif]

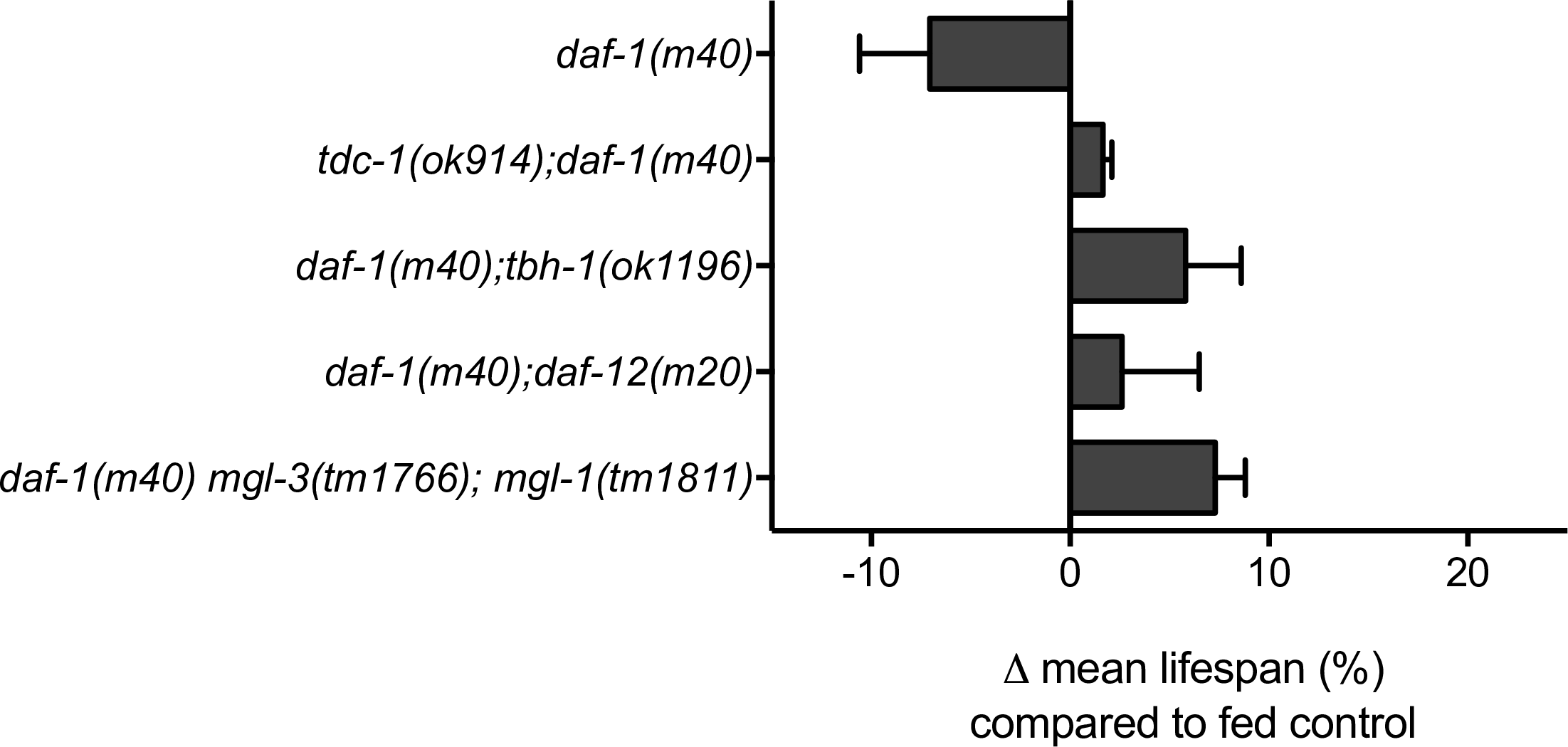

Supplement: S2 Fig — Summary of lifespan experiments suppressing specific phenotypes caused by increased daf-3 activity. Introducing suppressor mutations in genes controlling changes to feeding rate (tdc-1 and tbh-1), dauer formation (daf-12), and fat storage (mgl-3;mgl-1) was insufficient to restore lifespan extension by BD in daf-1(m40) mutant animals. * indicates BD lifespan was significantly different (p ≤ 0.001) than fed control group in all experiments, error bars reflect SEM. See S2 Table for individual experiment details. (TIF) [file pgen.1006544.s002.tif]

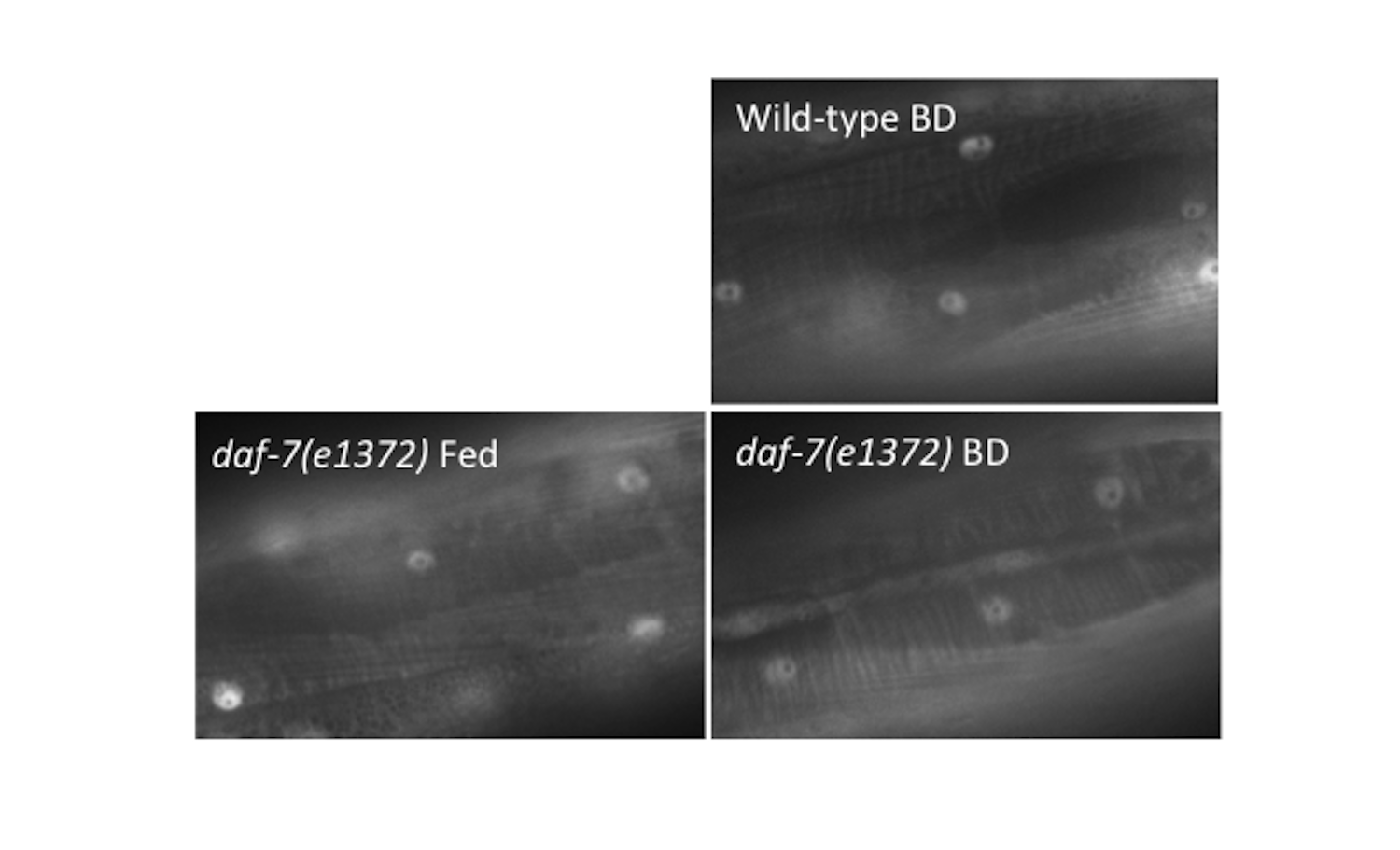

Supplement: S3 Fig — Representative images of the zIs356[daf-16p::daf-16a/b::GFP] reporter in muscle cells of BD treated wild-type or daf-7(e1372) animals in fed and BD conditions. (TIF) [file pgen.1006544.s003.tif]

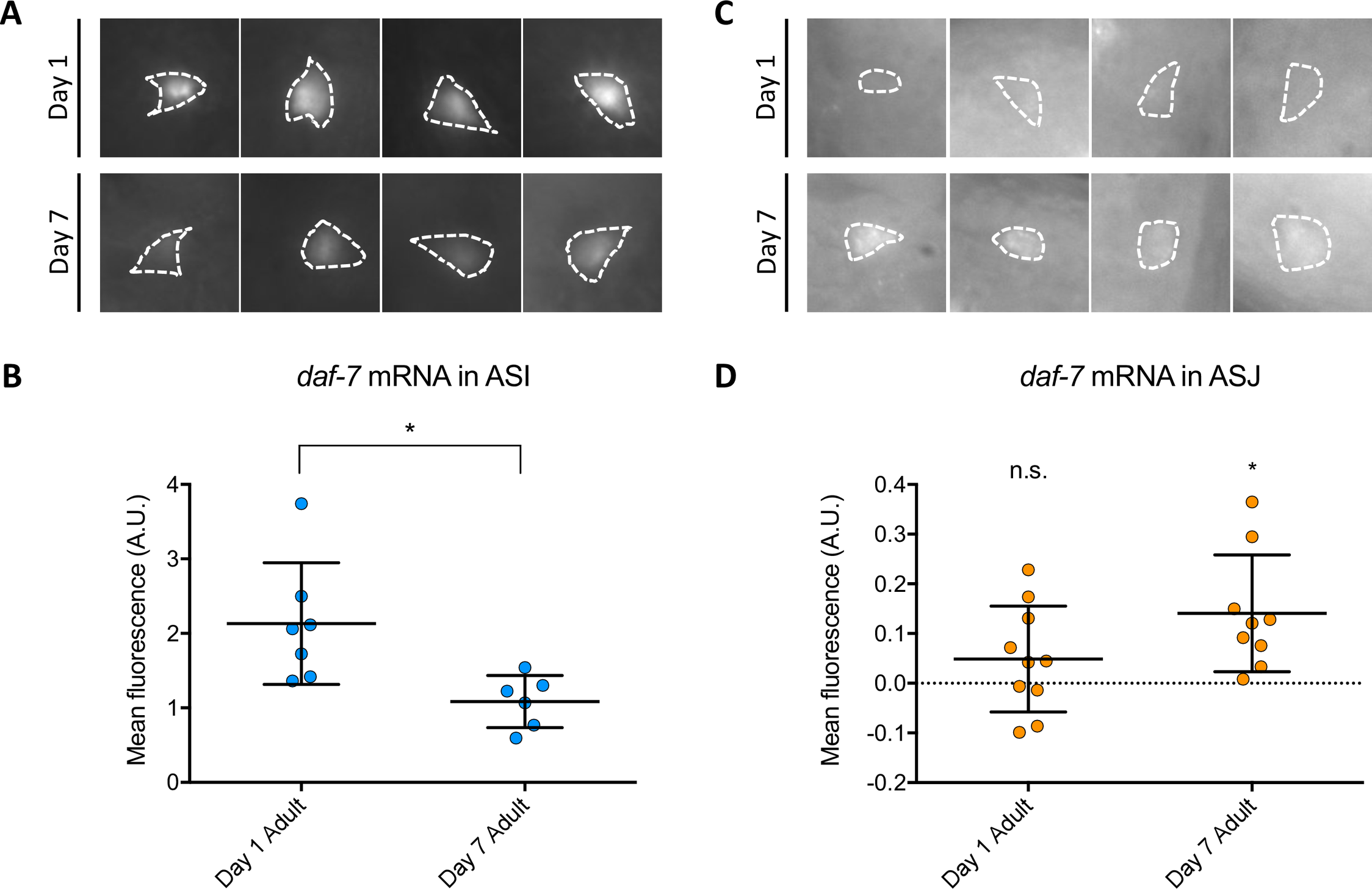

Supplement: S4 Fig — Images and quantification of fluorescence of FISH probes designed against daf-7 in ASI (identified by co-localization with str-3p::GFP) (A,B) and ASJ (identified by co-localization with trx-1p::GFP) (C,D) in young and aged animals. (B) * indicates p < 0.05 by unpaired t-test. (D) * indicates p < 0.05 significantly different from zero by one-sample t-test. (TIF) [file pgen.1006544.s004.tif]

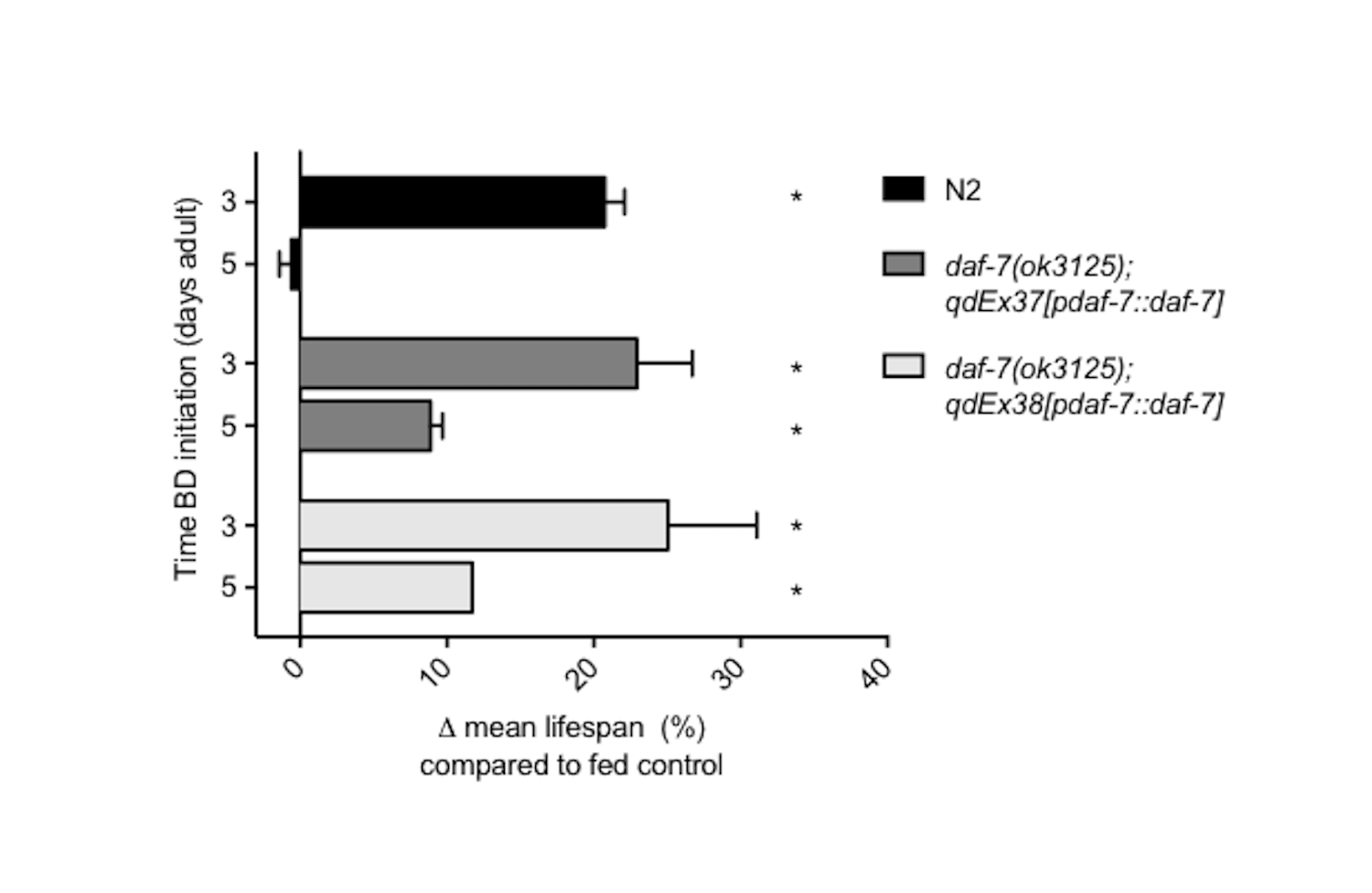

Supplement: S5 Fig — Summary of lifespan experiments in two rescue lines overexpressing daf-7. * indicates BD lifespan was significantly different (p ≤ 0.001) than fed control group in all experiments, error bars reflect SEM. See S4 Table for individual experiment details. (TIF) [file pgen.1006544.s005.tif]
